# Supplementary material for: Fermented dairy foods consumption and depressive symptoms: A meta-analysis of cohort studies
Source: PLoS One. 2023 Feb 6;18(2):e0281346. doi: 10.1371/journal.pone.0281346 (PMC9901789; doi:10.1371/journal.pone.0281346)
Supplement: Attachment — Submitted filename: Response to Reviewers.doc [file pone.0281346.s002.doc]

Dear Editor,

Thank you for your letter dated December 13, 2022 concerning our manuscript entitled “Fermented dairy foods consumption and depressive symptoms: a meta-analysis of cohort studies” (Manuscript ID **PONE-D-22-29070**). We would like to thank the anonymous reviewers and the editor for their constructive comments on revising this manuscript. According to the comments of the reviewers, we have revised the related part in the original manuscript and the revised portions were marked in red bold. The following is the correspondence to the reviewers concerning the comments and suggestions about the manuscript.

**Answers to the reviewer 1**

***1. Line 20, please define the WHO acronym.***

Answer: thanks for the reviewer’s comments, we have defined the WHO acronym.

***2.*** ***Please describe what you mean by "highest fermented dairy products." (describe the type of dairy products considered as highest fermented dairy products)***

Answer: thanks for the reviewer’s comments, we have revised the relating sentences.

***3.*** ***In the section "Evaluation heterogeneity," please clarify or discuss the meaning of I2 and Tau2.***

Answer: thanks for the reviewer’s comments. To explore sources of heterogeneity across studies, subgroup analysis was performed (P heterogeneity ＜ 0.001, I2 =76.7%, Tau2 = 0.0365 for higher yogurt intake, P heterogeneity = 0.532, I2 = 0%, Tau2 ＜ 0.001 for higher cheese intake; P heterogeneity ＜ 0.001, I2 =76.9%, Tau2 = 0.0615 for highest yogurt intake, P heterogeneity = 0.110, I2 = 46.9%, Tau2 = 0.0153 for highest cheese intake). Furthermore, we assessed publication year, region, follow-up period, disease type, and dairy type, number of cases, number of controls, morbidity of cases, and morbidity of controls. Finally, logistic meta-regression analyses revealed that morbidity of cases and morbidity of controls could substantially influence the initial heterogeneity.

***4.In the section "Sensitivity analysis," please include the p-value for the lack of significance.***

Answer: thanks for the reviewer’s comments. The results of sensitivity analysis were listed as follows:

***5.Why did you include data from fermented soybeans products (Table 1)? When this product is not a fermented dairy product. Please exclude this information from your meta-analysis.***

Answer: thanks for the both reviewers’ comments, we have re-analyzed the data.

**Answers to the reviewer 2**

***1.*** ***The authors declared that they included studies that assessed yogurt and cheese intake, but they also included a study with fermented soybeans.***

Answer: thanks for the both reviewers’ comments, we have re-analyzed the data.

***2.*** ***The exclusion criteria and PICO framework did not note.***

Answer: We evaluated the eligible studies if all the following conditions were met: (1) evaluation of the association of depression risk with yogurt or cheese consumption; (2) diet was generally assessed by food frequency questionnaire; (3) ORs and 95% CIs can be acquired or calculated from sufficient data in the manuscript or supplementary materials; (4) the publication was a cohort study; and (5) the study was published in English.

***3. The Eggers test can be used to check for publication bias..***

Answer: thanks very much for the reviewer’s suggestion, the Eggers test and Begg’s test were performed, all the value were above 0.05.

***4. The quality of the included studies should be assessed.***

Answer: We evaluated the eligible studies if all the following conditions were met: (1) evaluation of the association of depression risk with yogurt or cheese consumption; (2) diet was generally assessed by food frequency questionnaire; (3) ORs and 95% CIs can be acquired or calculated from sufficient data in the manuscript or supplementary materials; (4) the publication was a cohort study; and (5) the study was published in English.

***5. The authors should explain the cut-off values for higher intake and highest intake in more detail.***

Answer: the details of cut-off values were listed in table 1.The limitation of unified cutoff value for the fermented dairy foods consumption in the enrolled studies may also potentially affect the results was listed in the discussion.

***6. Please add the results of the meta-regression.***

Answer: thanks very much for the reviewer’s comment, we have added the results of subgroup analysis.

***7. Why were yogurt and yogurt drinks reported separately in the forest plot?***

***Adjusted variables and age are added to Table 1.***

***There are four duplicate studies in the present meta-analysis. The recent study with a larger sample size should be selected and included in the meta-analysis.***

Answer: thanks very much for the reviewer’s comment, yogurt and yogurt drinks are different type of fermented dairy foods, thus we reported it separately.

Thanks very much for the reviewer’s comment, adjusted variables are added to Table 1.

Thanks very much for the reviewer’s comment, we have searched the relating website with the inclusion criterion, and eight studies were available recently.

At this time, we address the statement that:

***The authors received no specific funding for this work.***

Thanks the anonymous reviewers and the editor again.

Sincerely Yours authors

December 19, 2022
